# Combined Therapy of Vitamin D3-Tolerogenic Dendritic Cells and Interferon-β in a Preclinical Model of Multiple Sclerosis

**DOI:** 10.3390/biomedicines9121758

**Published:** 2021-11-24

**Authors:** Bibiana Quirant-Sánchez, María José Mansilla, Juan Navarro-Barriuso, Silvia Presas-Rodríguez, Aina Teniente-Serra, Federico Fondelli, Cristina Ramo-Tello, Eva Martínez-Cáceres

**Affiliations:** 1 Immunology Division, LCMN, Germans Trias i Pujol University Hospital and Research Institute, Campus Can Ruti, 08916 Badalona, Spain; bquirant.germanstrias@gencat.cat (B.Q.-S.); mjmansilla@igtp.cat (M.J.M.); juan_navarro@outlook.com (J.N.-B.); ateniente.germanstrias@gencat.cat (A.T.-S.); ffondelli@igtp.cat (F.F.); 2 Department of Cellular Biology, Physiology and Immunology, Campus Bellaterra, Universitat Autònoma de Barcelona, 08193 Cerdanyola del Vallès, Spain; 3 Multiple Sclerosis Unit, Department of Neurosciences, Germans Trias i Pujol University Hospital, 08916 Badalona, Spain; spresas@igtp.cat; 4 Department of Medicine, Campus Bellaterra, Universitat Autònoma de Barcelona, 08193 Cerdanyola del Vallès, Spain

**Keywords:** combined therapy, antigen-specific therapies, immunomodulatory, multiple sclerosis

## Abstract

Autologous antigen-specific therapies based on tolerogenic dendritic cells (tolDC) offer the possibility to treat autoimmune diseases by restoring homeostasis and targeting specifically autoreactive responses. Here, we explore the hypothesis that systemic inflammation occurring in autoimmune diseases, such as multiple sclerosis (MS), can generate a disease-specific environment able to alter the functionality of tolDC. In this context in fact, a combined therapy of tolDC with an immunomodulatory treatment could potentiate the beneficial effect of this antigen-specific cell therapy. For this purpose, we analyzed the efficacy of a combined therapy based on the use of vitamin D3 (VitD3)-tolDC plus interferon beta (IFN-beta) in MS. VitD3-tolDC were generated from healthy donors and MS patients and co-cultured with allogeneic peripheral blood mononuclear cells, in the presence or absence of IFN-beta. In vitro, VitD3-tolDC treatment reduced the percentage of activated T cells and allogeneic proliferation, whereas VitD3-tolDC+IFN-beta treatment enhanced the suppressive ability of VitD3-tolDC and, additionally, induced a shift towards a Th2 profile. To determine the clinical benefit of the combined therapy, C57BL/6-experimental autoimmune encephalomyelitis (EAE)-induced mice were treated with antigen-specific VitD3-tolDC and/or IFN-beta. Treatment of EAE mice with combined therapy ameliorated the disease course compared to each monotherapy. These results suggest that a combined therapy based on antigen-specific VitD3-tolDC and IFN-beta may represent a promising strategy for MS patients.

## 1. Introduction

Tolerance is a state of unresponsiveness of the immune system towards antigens that can elicit an immune response. It is a mechanism primarily aimed to avoid the reactivity of the immune system against “self” tissues. Indeed, when tolerance towards self-antigens is lost, autoimmune diseases, such as multiple sclerosis (MS), can develop [1]. 

MS is the most common multifocal inflammatory demyelinating disease of the central nervous system (CNS). Diagnosis of MS is based on clinical, radiological, and laboratory criteria (oligoclonal bands in the cerebrospinal fluid). Currently, available therapies for MS exert their effects through immunomodulatory or immunosuppressive functions. These treatments are focused on reducing the activity of the disease and slowing its progression, but it is a disease that has no cure [2].

MS is considered an immune-mediated disease. Extensively reported in both healthy individuals and MS patients is the presence of circulating myelin-specific autoreactive T cells. However, the specific mechanisms causing their activation and infiltration of CNS, resulting in wide systemic inflammation and neurodegeneration, are still unknown [3,4,5]. In MS patients, altered and dysfunctional regulatory T cells (Treg), monocytes and dendritic cells (DC), have been described [6,7]. In this scenario, DC are pivotal regulators of the immune response, able to induce T cell priming and differentiation [5], control T effector cells and promote Treg differentiation in the periphery. The presence of dysfunctional Treg [3], DC [5] and altered cytokine production may facilitate the activation and arrival of autoreactive T cells into the CNS [3,8,9]. 

Recombinant interferon beta (IFN-beta) is one of the most prescribed treatments for relapsing-remitting MS (RRMS) patients. This is due to its multi-level immunomodulatory and anti-inflammatory functionality: the inhibition of Th1 response, reduction in T cell expansion, decrease in costimulatory and MHC class-II molecules expression, induction of Th2 cytokine profile, and blockage of immune cells entry into the CNS [10,11,12,13]. Because of the long experience using IFN-beta for treating MS patients and its wide immunomodulatory effects, we aim to demonstrate that the combination of myelin-specific vitamin D3 tolerogenic dendritic cells (VitD3-tolDC) with IFN-beta could improve the therapeutic effect of myelin-specific VitD3-tolDC. 

Antigen-specific cell-based tolerogenic therapies (CTT) are promising therapeutic strategies [14,15,16] that, in contrast to the currently available immunomodulatory or immunosuppressive drugs, have the potential to restore self-tolerance by suppressing the autoreactive T cell clones [17,18,19,20,21]. In this context, our research group has developed an autologous cell-based tolerogenic product consisting of monocyte-derived tolerogenic DC generated in the presence of vitamin D3 (VitD3-tolDC) and loaded with a pool of myelin peptides [22]. Preclinical data in experimental autoimmune encephalomyelitis (EAE) demonstrated that the treatment with antigen-specific VitD3-tolDC is able to abrogate disease progression when they were administrated to mice with clinical symptoms of the disease [23,24,25]. Currently, two harmonized phase I clinical trials using autologous myelin-specific VitD3-tolDC therapy in patients with active forms of MS are ongoing in Spain and Belgium [22,26]. 

However, the potent inflammatory processes taking place in MS patients may influence the phenotype and functionality of the VitD3-tolDC administrated. In order to get an efficient translation of this therapy to the clinic, we hypothesize that the combination of antigen-specific VitD3-tolDC with an immunomodulatory drug, could act synergically by restoring an appropriate immune environment, potentiating the antigen-specific tolerogenic effect of VitD3-tolDC.

## 2. Materials and Methods

**Figure 1:**
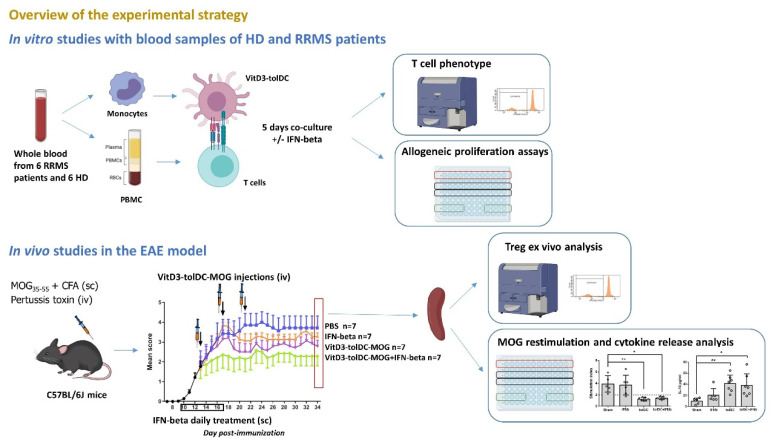
Overview of the experimental strategy. Two experimental approaches were used to investigate the effect of the combined VitD3-tolDC plus IFN-beta therapy in MS. In vitro studies: we performed in vitro co-cultures of VitD3-tolDC plus PBMC of RRMS patients and of HD (*n* = 6/group) in presence/absence of IFN-beta. After 5 days of culture, the phenotype of cultured T cells and the induction of allogeneic proliferation by different concentrations of VitD3-tolDC in presence/absence of IFN-beta were analyzed (see Methods for a more detailed explanation); in vivo studies: EAE was induced to C57BL/6J mice. Mice were treated subcutaneously (sc) with IFN-beta (from day 10 to 17 post-immunization, (pi)) and /or intravenously (iv) with antigen-specific VitD3-tolDC (VitD3-tolDC-MOG) (on days 13, 17 and 21 pi) (*n* = 7/group). Clinical scores were recorded daily. Splenocytes of mice (*n* = 7/group) were analyzed to evaluate antigen-specific proliferation (ex-vivo restimulation with MOG antigen), secretion of cytokines and percentage of regulatory T cells (Treg). * *p* < 0.05, ** *p* < 0.01.

An overview of the experimental strategy is represented in Figure 1.

### 2.1. Sample Collection 

Monocyte-derived dendritic cells (MDDC) differentiation was performed from 6 buffy coat samples obtained following standard operating procedures for blood donation, provided by the Banc de Sang i Teixits (Barcelona, Spain). In addition, 40 mL of peripheral blood samples from 6 Relapsing Remitting MS patients (RRMS) from the MS unit of the Germans Trias i Pujol Hospital (Spain) were collected in lithium heparin tubes and processed as detailed below. Patients selected did not receive any treatment during at least the previous two months (Supplementary Table S1). The study was approved by the Ethical Committee of Germans Trias i Pujol Hospital and all subjects gave their informed consent according to the Declaration of Helsinki. 

### 2.2. Monocyte Isolation

To isolate monocytes from RRMS patients, peripheral mononuclear cells (PBMC) from 40 mL whole blood samples were obtained by ficoll-hypaque gradient (Rafer, Zaragoza, Spain) and positive selection of CD14+ cells was performed using the EasySep uman CD14 Positive Selection kit (StemCell Technologies, Vancouver, BC, Canada) following the manufacturer’s instructions. In the case of buffy coats, a depletion of T lymphocytes (CD3+ cells) was performed incubating samples with RosetteSep Human CD3 Depletion Cocktail (StemCell Technologies) before ficoll-hypaque gradient separation due to the high number of cells present in the buffy coat samples.

Samples were incubated with 7-amino-actinomycin D (7-AAD) (BD Biosciences, San José, CA, USA) and annexin V-PE (Immunotools, Friesoythe; Germany) for 20 min at 4 °C in darkness to analyze the viability of monocytes. In addition, perfect count microspheres (Cytognos S.L, Salamanca, Spain) were added to each sample and acquired in a flow cytometer (FACS Canto II, BD Biosciences) to determine the number of viable monocytes obtained.

### 2.3. The Generation of Vitamin D3-Tolerogenic Dendritic Cells

Monocytes were cultured for 6 days in X-VIVO 15 medium (Lonza, Basel, Switzerland) supplemented with 100 U/mL penicillin (Reig Jofre, Sant Joan Despí, Spain) and 100 µg/mL streptomycin (Normon, Tres Cantos, Spain) + 2 nM L-Glu (Sigma-Aldrich, St Louis, MI, USA) + 2% human AB serum, in the presence of 200 U/mL granulocyte-macrophage colony-stimulating factor (Peprotech, London, UK) and 250 U/mL interleukin 4 (Peprotech) for 6 days, as previously reported [27]. VitD3-tolDC and mDC were treated on day 4 with a maturation cocktail of cytokines containing 1000 U/mL tumor necrosis factor alpha (Peprotech), 1000 U/mL IL-1β (Peprotech) and 1 µM prostaglandin E2 (Pfizer, New York, NY, USA). While mDC did not receive any additional stimuli, VitD3-tolDC were obtained by adding 1 nM vitamin D3 (Calcijex, Abbott, Chicago, IL, USA) on days 0 and 4. On day 6, cells were harvested after 30 min of incubation with accutase (Invitrogen, San Diego, CA, USA) and washed twice. Cell viability and count were determined by flow cytometry (as mentioned above). Functional and phenotypical characterizations were performed as previously reported [27]. Briefly, to determine the stimulatory capacity of DC, either 5000 immature DC (iDC), mature DC (mDC), or VitD3-tolDC, were incubated in X-VIVO 15 medium with 1 × 10^5^ allogeneic PBMC. Six replicated wells of each condition were performed. Allogeneic proliferative assays were performed for 5 days at 37 °C in a 5% CO_2_ atmosphere. During the last 18 h, 1 μCi of [3H]-methylthymidine (PerkinElmer, Waltham, MA, USA) was added to each well. Cells were harvested onto glass fiber filters, and [3H]-methylthymidine incorporation was measured in a β-scintillation counter (Trilux, Wallac, Ramsey, MN, USA). 

### 2.4. Allogeneic Proliferative Response

PBMC were isolated from whole blood by ficoll-density gradient centrifugation. Cell viability and counts were performed following the protocol described above.

MDDC were harvested on day 6 of differentiation and co-cultured with allogenic PBMC (2 × 10^5^ PBMC co-cultured with 5 × 10^3^ MDDC) for 5 days in supplemented X-VIVO 15 medium, in the presence or absence of 10 IU/mL IFN-beta 1a (Rebif^®^, Merck Serono Darmstadt, Germany).

PBMC were co-cultured with different percentages of VitD3-tolDC: (a) 5.10^3^ VitD3-tolDC (100% VitD3-tolDC) condition; (b) 3,75.10^3^ VitD3-tolDC and 1,25.10^3^ mDC (75% VitD3-tolDC) condition; (c) 2,5.10^3^ VitD3-tolDC and 2,5.10^3^ mDC (50% VitD3-tolDC) condition; and (d) 1,25.10^3^ VitD3-tolDC and 3,75.10^3^ mDC (25% VitD3-tolDC) condition, in the presence/absence of 10 IU/mL of IFN-beta. After 5 days of culture, proliferation of PBMC was analyzed by [3H]-methyl-thymidine incorporation. The values of PBMC co-cultured with 5.10^3^ mDC were used as positive control.

### 2.5. Phenotype 

#### 2.5.1. The Phenotype of Monocyte-Derived Dendritic Cells 

Cells from each culture condition were incubated with monoclonal antibodies for 20 min at room temperature in the dark. The following monoclonal antibodies were used: CD11c PE-Cy7, CD14 V450, CD83 APC, CD86 FITC and HLA-DR V500 (BD Bioscience). At least 10,000 CD11c+ cells of each condition were acquired on a FACS Canto II flow cytometer (BD Biosciences). 

#### 2.5.2. The Phenotype of Peripheral Blood Mononuclear Cells

After 5 days of culture, PBMC co-cultured with mature DC (mDC), VitD3-tolDC (100% VitD3-tolDC) and 50% VitD3-tolDC (50% VitD3-tolDC + 50% mDC) in the presence and absence of IFN-beta were harvested and stained using CD3-V450, CD4-PerCP-Cy5.5, CD45RA PE-Cy7, CCR7 PE, CD38 APC, CD8 APC-H7, HLA-DR V500 (BD Bioscience), CD183 AF488, CD196 BV605 and CD45 AF700 (Biolegend, San Diego, CA, USA) for T cell analysis; and CD4 PerCP-Cy5.5, CD25 PE, CCR4 PE-Cy7, CD127 AF647, CD45RO APC-H7, CD3 V450, HLA-DR V500 (BD Biosciences) and CD45 AF700 (Biolegend) for Treg analysis. Monoclonal antibodies were incubated for 20 min at room temperature and protected from the light. Samples were washed and a total of 50,000 CD3+ events were acquired on an LSR Fortessa flow cytometer (BD Biosciences). Both panels were analyzed using FACSDiva software (BD Biosciences). The gating strategy used to analyze the desired T cell subpopulations was previously reported [28].

#### 2.5.3. The Phenotype of Splenocytes from C57BL/6J Mice

The percentage of Treg from ex vivo splenocytes was analyzed following the manufacturer’s instructions. Briefly, 5 × 10^5^ splenocytes were stained with anti-CD3, CD4, CD25 and intracellular FoxP3 (all from BD Pharmingen, San Diego, CA, USA), and analyzed in a FACS Canto II and FACS Diva software (BD Biosciences).

### 2.6. Animals

Female C57BL/6J mice, 8–10 weeks old, were purchased from Envigo Rms Spain SL (Sant Feliu de Codines, Barcelona, Spain) and housed at the Comparative Medicine and Bioimage Centre of Catalonia (CMCiB) under standard light- and climate-controlled conditions, with standard chow diet and water provided ad libitum.

### 2.7. Bone Marrow-Derived Dendritic Cells Generation

BMDC were generated following the protocol previously described by Mansilla et al. in 2015 and 2016 [24,25]. Briefly, progenitor bone marrow cells were obtained from C57BL/6 donor mice and cultured in RPMI medium containing 1000 IU/mL of murine GM-CSF (Peprotech). VitD3-tolDC were generated by adding 1 nM 1α, 25-dihydroxyvitamin D3 (Kern Pharma, Terrassa, Spain) for 8 days. On day 7, 0.1 mg/mL lipopolysaccharide (LPS; Sigma-Aldrich) was added to the culture medium of mDC and VitD3-tolDC. After 22–24 h of LPS stimulation, DC were pulsed with 10 μM the peptide 35-55 of myelin oligodendrocyte glycoprotein (MOG_35-55_) for 18 h. Finally, VitD3-tolDC-MOG were cryopreserved in aliquots of 10^7^ cells and stored in liquid nitrogen until use.

VitD3-tolDC were characterized by analyzing their phenotype (the reduction in the expression of the co-stimulatory molecules CD80 and CD40; as well as MHC II) and functionality (suppression of T cell proliferation in co-cultures of DC with allogeneic splenocytes) to ensure their correct production. 

### 2.8. Induction of EAE and Clinical Follow-Up

For EAE induction, mice were anesthetized with ketamine/xylazine at 50 and 5 mg/kg body weight, respectively, and then immunized subcutaneously with 100 μg of MOG_35–55_ (YRSPFSRVVHLYRNGK) (Immunostep, Salamanca, Spain), emulsified in an equal volume (1:1) of Freund’s complete adjuvant containing 4 mg/mL of *Mycobacterium tuberculosis* (strain H37RA, Difco, Detroit, MI, USA). In addition, 250 ng of pertussis toxin (Sigma Chemical, St. Louis, MO, USA) was injected intravenously (iv) at day 0 and 2. All animals were weighed and examined daily for welfare and clinical signs. Clinical evaluation was performed in a blinded manner by two different observers according to the following criteria: 0, asymptomatic; 0.5, loss of distal half of tail tone; 1, loss of entire tail tone; 1.5, hind limb weakness; 2, hind limb paralysis; 2.5, hind limb paraplegia; 3, forelimb weakness; 4, quadriparesis; 4.5, severe quadriparesis; 5, quadriplegia; and 6, death. Endpoint criteria were established to minimize suffering and ensure animal welfare.

### 2.9. In Vivo Treatment of EAE Mice

C57BL/6-EAE induced mice were treated daily from day 10 to 17 post-immunization (pi) with IFN-beta (5000 IU of IFN-beta1a, subcutaneous, sc) and/or on day 13, 17 and 21pi with VitD3-tolDC-MOG (1 × 10^6^ cells, iv) (*n* = 7 mice/group) and monitored for 34 days. The vehicle group received 200 μL of both sc and iv administration of PBS.

### 2.10. Antigen-Specific T Cell Reactivity and Cytokine Secretion

Mice were euthanized on day 34 pi, and splenocytes from the vehicle (PBS), VitD3-tolDC-MOG, IFN-beta, or VitD3-tolDC-MOG+ IFN-beta groups were cultured in a 96-well plate at 2 × 10^5^ cells/well in 200 μL of supplemented RPMI containing 5 μM MOG_35-55_, 5 μM of phytohemagglutinin (PHA) (Sigma-Aldrich) (positive control) or culture medium (negative control). After 48 h of culture, 50 μL of supernatant of each well was collected and stored for cytokine release detection, and 1 μCi/well of [3H]-thymidine (PerkinElmer) was added for the last 18 h of culture. The stimulation index (SI) for each stimulus was calculated as the mean counts per minute (cpm) of antigen-stimulated cultures divided by the mean cpm of the non-stimulated cultures.

The secretion of IL-2, IL-4, IL-6, IFN-γ, TNF-α, IL-17, and IL-10 cytokines were quantified in the culture supernatants using the BD cytometric bead array mouse Th1/Th2/Th17 cytokine kit (BD Biosciences) and an LRS Fortessa cytometer (BD Biosciences) according to the manufacturer’s instructions.

### 2.11. Statistical Analysis

Statistical analyses were performed using GraphPad Prism version 6.0 for Windows (La Jolla, CA, USA). Parametric and nonparametric tests were used depending on the normality distribution of the variables. To compare data from two groups, Mann–Whitney U or t tests were applied. When more than two groups were compared, non-parametric one-way ANOVA (Kruskal–Wallis) followed by Dunnett’s multiple comparisons tests were applied. Differences were considered statistically significant when *p* <  0.05. Data were expressed as the mean ± standard deviation (SD) values unless otherwise stated.3. 

## 3. Results

### 3.1. The Combination of VitD3-tolDC and IFN-Beta Treatment Enhances the Suppressive Ability of VitD3-tolDC and IFN-Beta Monotherapies 

First, the optimal concentration of IFN-beta to evaluate the anti-proliferative property of this cytokine on peripheral PBMC cultures was analyzed. To do so, 0.2 × 10^6^ PBMC stimulated to proliferate with mitogens (25 ng/mL PMA+ 250 ng/mL Ionomycin), were cultured in presence of 10 or 100 IU/mL of IFN-beta. After 5 days of culture, the inhibition of proliferation was measured compared to the reference condition (PBMC stimulated with mitogens without IFN-beta treatment). Our results showed a suppression of 29.39 ± 13.58% (10 IU/mL of IFN-beta) and of 18.60 ± 9.67% (100 IU/mL of IFN-beta) (*n* = 3 for each condition). Based on these results, a concentration of 10 IU/mL of IFN-beta was selected to evaluate the IFN-beta effect on the immune function.

The ability of VitD3-tolDC to suppress allogeneic PBMC proliferation was evaluated for VitD3-tolDC generated from RRMS patients and HD (*n* = 6 for each group). VitD3-tolDC induced a suppression of allogeneic proliferation of 54.73 ± 9.90% in PBMC of RRMS patients and of 56.14 ± 18.43% in PBMC of HD in relation to the reference control mDC. On the other side, no statistical differences were observed in the suppressive capability among VitD3-tolDC generated from RRMS patients and HD (Table 1). 

**Figure 2:**
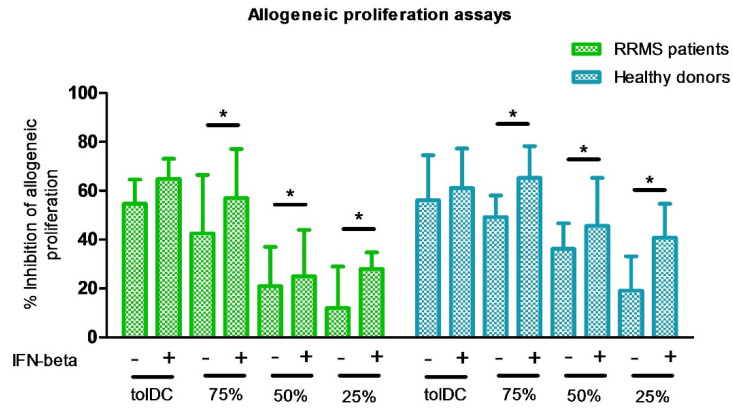
Dose-dependent reduction of allogeneic proliferation of PBMC, cultured with different percentages of VitD3-tolDC in presence/absence of IFN-beta. Suppressive ability of VitD3-tolDC on allogeneic PBMC from RRMS patients (green) and HD (blue) is represented as the percentage of proliferation compared to mature DC (mDC) stimulus. Different percentages of VitD3-tolDC and mDC are represented: 100% (100% VitD3-tolDC), 75% (75% VitD3-tolDC + 25% mDC), 50% (50% VitD3-tolDC + 50% mDC) and 25% (25% VitD3-tolDC +75% mDC) in presence/absence of IFN-beta. Error bars correspond to SEM; * *p* < 0.05.

Then, PBMC were co-cultured with different percentages of VitD3-tolDC, as specified in Methods (Section 2.4), in the presence/absence of 10 IU/mL of IFN-beta. The percentage of inhibition of allogeneic proliferation, in relation to the reference control (mDC) was analyzed. Results obtained show a dose dependent suppressive effect of VitD3-tolDC. Addition of 10 IU/mL IFN-beta increased the suppressive effect of VitD3-tolDC (Figure 2 and Table 1).

### 3.2. Treatment with VitD3-tolDC Reduces the Activation of T Lymphocytes from RRMS Patients

**Figure 3:**
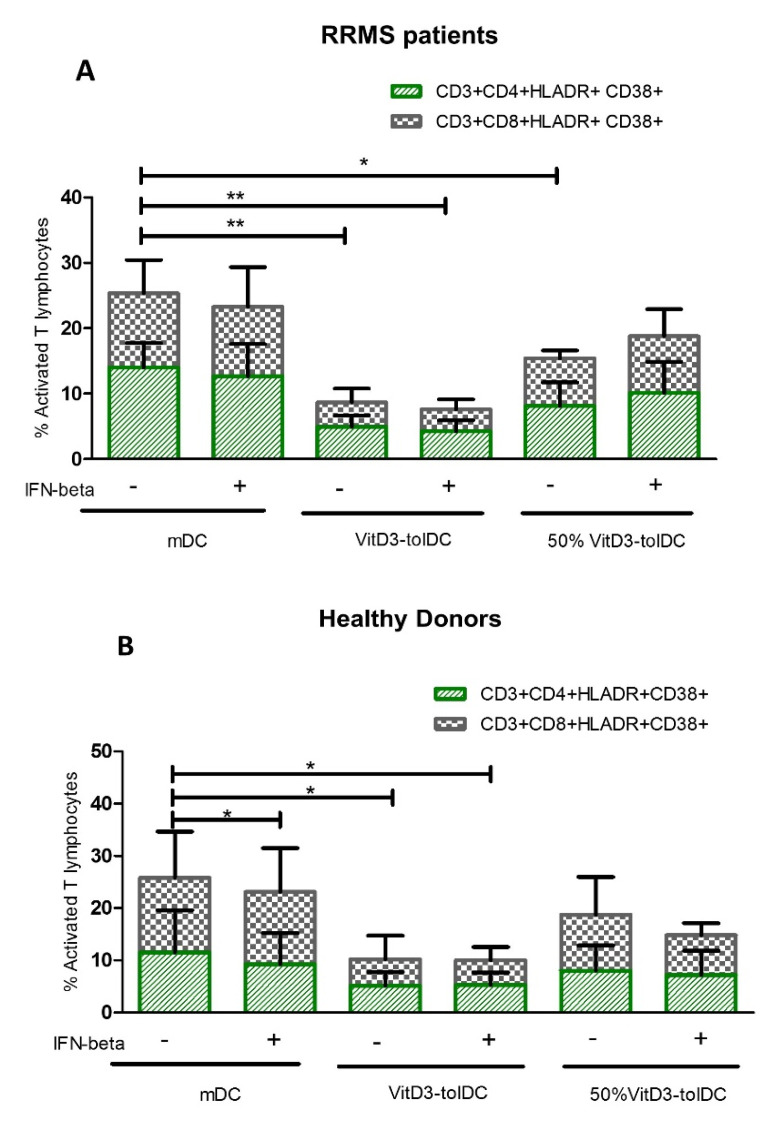
VitD3-tolDC reduce the activation of T lymphocytes in RRMS patients and HD. The percentage of activated CD4 + lymphocytes (CD3+CD4+HLA-DR+CD38+) (green) and CD8+ lymphocytes (CD3+CD8+HLA-DR+CD38+) (gray) in co-cultures of PBMC with mature DC (mDC), (100% VitD3-tolDC) or (50%VitD3-tolDC), in presence/absence of IFN-beta was analyzed in relapsing-remitting multiple sclerosis (RRMS) patients (**A**) and healthy donors (HD) (**B**) (*n* = 6 each group). Data represent the relative percentage of each lymphocyte subpopulation n relation to total percentages of CD4+ or CD8+ T cells. Error bars correspond to SEM; * *p* < 0.05, ** *p* < 0.01.

To further investigate the in vitro effect of VitD3-tolDC and IFN-beta combined therapy on lymphocytes, the phenotype of PBMC co-cultured for 5 days with two concentrations of VitD3-tolDC (100% VitD3-tolDC and 50% VitD3-tolDC) in the presence/absence of IFN-beta was determined and compared to the reference condition (mDC). As shown in Figure 3, the presence of 100%VitD3-tolDC in PBMC cultures, reduced the percentage of activated CD4+ and CD8+ T cells (HLA-DR+ CD38+ T lymphocytes) in both, RRMS patients and HD, compared to the reference condition (mDC) (*p* < 0.05), except for activated CD4+ T cells from HD (*p* = 0.050) (*n* = 6 for each group) (Table 2). However, no differences in phenotype were observed with the addition of IFN-beta to PBMC cultures in any of the culture conditions, except for activated CD8+ T cell in HD (Figure 3 and Table 2).

No statistical differences were found in the analysis of the maturation stages (naïve, central memory, effector memory and effector compartments) of either CD4+ or CD8+ T cells (Supplementary Table S2) in the different culture conditions analyzed. 

### 3.3. VitD3-tolDC+IFN-Beta Culture Induces the Differentiation of Th2 Lymphocytes 

**Figure 4:**
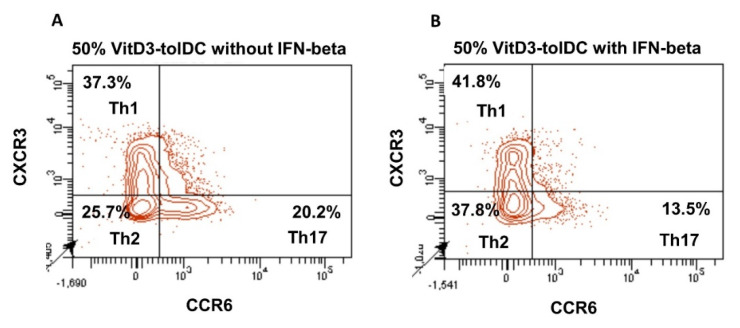
Immunomodulatory effect of VitD3-tolDC with/without IFN-beta on T lymphocytes. Representative example of changes in the relative percentages of Th1, Th2 and Th17 lymphocytes in relation to total CD4+ T lymphocytes, after 5 days co-culture of 50% VitD3-tolDC in absence (**A**) or presence (**B**) of IFN-beta.

The effect of VitD3-tolDC in presence/absence of IFN-beta on different functional stages of lymphocytes was studied. Percentages of Th1 (CD3+ CD4+ CXCR3+ CCR6-), Th2 (CD3+ CD4+ CXCR3- CCR6-), Th17 (CD3+ CD4+ CXCR3- CCR6+) and Treg (CD4+ CD25+ CCR4+ CD45RO+) cells were analyzed in PBMC cultures from RRMS patients and HD, after 5 days of co-culture. A representative experiment is shown in Figure 4. 

**Figure 5:**
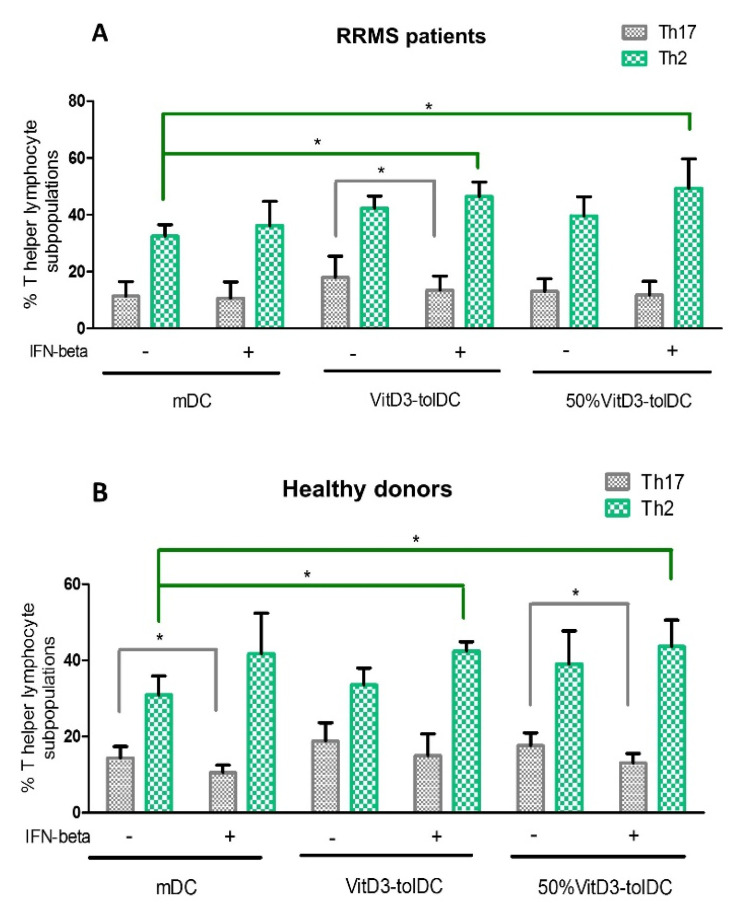
Effect of VitD3-tolDC in presence/absence of IFN-beta on the differentiation of Th2 and Th17 cells. Percentage of Th2 (CD3+ CD4+ CXCR3- CCR6-) lymphocytes (green) and Th17 (CD3+ CD4+ CXCR3- CCR6+) lymphocytes (gray) from (**A**) relapsing-remitting multiple sclerosis (RRMS) patients (*n* = 6) and (**B**) healthy donors (HD) (*n* = 6). The percentages of Th2 and Th17 lymphocyte subpopulations were analyzed on PBMC co-cultured with mature DC (mDC), 100%VitD3-tolDC or 50%VitD3-tolDC, in presence/absence of IFN-beta after 5 days of culture. Data represent the relative percentage of each lymphocyte subpopulation in relation to total CD4+ T cells. Error bars correspond to SEM; * *p* < 0.05.

As shown in Figure 5, we observed a significant increase in the percentage of Th2 lymphocytes when PBMC were co-cultured with 100%VitD3-tolDC+IFN-beta or 50%VitD3-tolDC+IFN-beta, compared to the reference condition (mDC) (*p* < 0.05), either in RRMS patients (Figure 5A) or HD (Figure 5B). In contrast, no significant differences were found when PBMC were co-cultured only with 100%VitD3-tolDC or 50%VitD3-tolDC (without IFN-beta) compared to mDC, either in RRMS patients or HD (Figure 5 and Supplementary Table S2).

When analyzing Th17 cells in RRMS patients, we only observed a significant reduction in this subpopulation when IFN-beta was added to PBMC co-cultured with 100%VitD3-tolDC (*p* < 0.05) (Figure 5A and Supplementary Table S2). In contrast, in HD, this decrease in Th17 cells was only significant when IFN-beta was added to PBMC co-cultured with mDC or 50%VitD3-tolDC (*p* < 0.05) (Figure 5B and Supplementary Table S2).

No differences in the percentage of Th1 and Treg were found in any of the different culture conditions analyzed (Supplementary Table S2).

### 3.4. Antigen-Specific VitD3-tolDC Administration in Combination with IFN-Beta Treatment Improves EAE Clinical Signs

**Figure 6:**
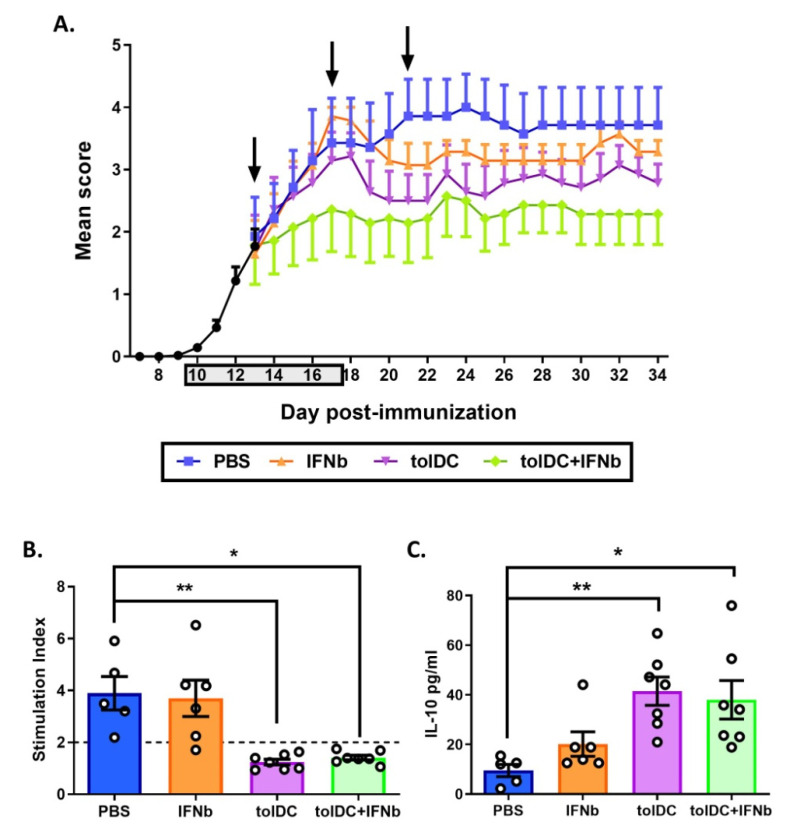
Combined therapy of antigen-specific VitD3-tolDC-MOG+IFN-beta ameliorates clinical signs of EAE. (**A**) Representation of daily mean clinical score of mice treated with vehicle (PBS) (blue), IFN-beta (IFNb) [5000IU] (orange), VitD3-tolDC-MOG [1 × 10^6^ cells] (tolDC) (violet) or VitD3-tolDC-MOG+IFN-beta (tolDC+IFNb) (green) (*n* = 7/group) for 34 days of follow-up. Gray square in the X axis and arrows indicate daily treatment period with IFN-beta (from day 10 to 17 post-immunization, pi) and VitD3-tolDC-MOG administration (days 13, 17 and 21 pi), respectively. (**B**) Analysis of antigen-specific T cell reactivity to MOG_35-55_ in splenocytes from mice treated with vehicle (PBS), IFN-beta (IFNb), VitD3-tolDC-MOG (tolDC) or VitD3-tolDC-MOG+IFN-beta (tolDC+IFNb) on day 34 pi. (**C**) Level of IL-10 in the supernatant of re-stimulated splenocytes with MOG_35-55_ antigen from each group of mice. Errors bars correspond to SEM. * *p* < 0.05, ** *p* < 0.01.

To investigate the in vivo efficacy of the administration of antigen-specific VitD3-tolDC in combination with IFN-beta treatment, VitD3-induced tolerogenic bone marrow DC loaded with the peptide 35-55 of myelin oligodendrocyte glycoprotein (MOG_35-55_) were generated VitD3-tolDC-MOG and administrated to mice with clinical signs of EAE under IFN-beta treatment. The clinical follow-up of mice receiving the combined therapy was compared to mice treated only with VitD3-tolDC-MOG, IFN-beta or vehicle (PBS). As shown in Figure 6A, while the treatment with IFN-beta did not significantly improve the disease severity of EAE mice compared to the vehicle group (mean clinical score at day 34 pi: 3.29 ± 0.49 for IFN-beta group and 3.71 ± 1.60 for PBS treated mice), the administration of antigen-specific VitD3-tolDC-MOG, both alone or in combination with IFN-beta treatment, caused an important reduction in the severity of the disease (mean clinical score at day 34 pi: 2.79 ± 0.81 for the VitD3-tolDC-MOG group and 2.29 ± 1.29 for the VitD3-tolDC-MOG+IFN-beta treated mice; *p* < 0.001 for both groups). Indeed, the beneficial effect of VitD3-tolDC-MOG therapy was significantly higher when this cell therapy was administered in combination with IFN-beta than alone (*p* < 0.05) (Figure 6A).

### 3.5. VitD3-tolDC-MOG Treatment Reduces Antigen-Specific T Cell Reactivity 

Mice treated solely with VitD3-tolDC-MOG or with the combination of VitD3-tolDC-MOG+IFN-beta exhibited reduced antigen-specific T cell reactivity compared to both, vehicle and IFN-beta group (*p* < 0.01 and *p* < 0.05, respectively) (Figure 6B). In contrast, the administration of IFN-beta alone, did not decrease T cell proliferation against MOG (Figure 6B). Moreover, when cytokine secretion profile of splenocytes re-stimulated with MOG_35-55_ antigen was analyzed, an increased IL-10 secretion was detected in the two groups of mice treated with VitD3-tolDC-MOG (VitD3-tolDC-MOG: *p* < 0.01 and VitD3-tolDC-MOG+IFN-beta: *p* < 0.05), but it was not enhanced by IFN-beta administration, neither in mice treated with IFN-beta vs. the vehicle group, nor mice receiving VitD3-tolDC-MOG+IFN-beta vs. the VitD3-tolDC-MOG group (Figure 6C). Regarding the ex vivo percentage of Treg in the spleen (in relation to total CD4+ splenocytes), no differences were found between any of the groups (data not shown).

## 4. Discussion

Despite the substantial advances in the understanding of MS pathology, none of the currently existing therapies are able to stop the course of the disease by inducing a long-term remission [18,29,30]. Nowadays, available treatments are based on a continuous dependence on nonspecific immunosuppressive or immunomodulatory drugs [31,32,33,34,35,36,37,38,39,40,41], exposing patients to potentially serious adverse effects, that may compromise their safety and adherence to therapy [42,43,44,45,46]. Fortunately, during the last decade, the increase in the knowledge of immune regulatory mechanisms has offered the possibility to claim antigen-specific CTT as a potential strategy to restore immune tolerance in patients with autoimmune diseases avoiding undesirable and potentially dangerous side effects [47,48,49]. 

Due to the complexity of MS pathology, a therapeutic approach based on a combination of treatments targeting different pathways involved in the induction and maintenance of tolerance may be required to fully control autoimmunity [50,51,52,53,54]. In this regard, several murine studies have demonstrated that administration of immunosuppressive drugs, such as dexamethasone or rapamycin, potentiate the regulatory effect of tolDC by prolonging allograft survival or by being beneficial in treating different autoimmune diseases [55,56,57,58,59,60]. In our approach, we evaluated the effect of combined VitD3-tolDC and IFN-beta treatment in the EAE model as well as using co-cultures of peripheral blood lymphocytes from MS patients. Our results showed that the combined therapy had an enhanced effect compared to each monotherapy in the above-mentioned models. Furthermore, we identified which lymphocyte subpopulations and functional subsets were specifically affected by VitD3-tolDC+IFN-beta treatment. 

Here, the in vitro experiments using MS and HD samples showed that VitD3-tolDC induces a decrease in CD4+ and CD8+ T cells activation. These results are in accordance with our previous studies reporting a strong antigen-specific hypo-responsiveness mediated by a down-modulation of genes involved in cell cycle and pro-inflammatory immune response [27,60]. Moreover, we found a low level of antigen-specific T cell reactivity in splenocytes from EAE mice treated with VitD3-tolDC-MOG, both in the presence and absence of IFN-beta treatment. In this regard, and in agreement with our previous data [24], splenocytes from EAE mice treated with VitD3-tolDC-MOG exhibited inhibition of antigen-specific T cell reactivity and an increase of IL-10 secretion when re-stimulated with MOG_35-55_ antigen. Consequently, in vivo results suggest that VitD3-tolDC are inducing a complex immunoregulation mediated by IL-10 that reduces T cell activation [25].

On the other hand, IFN-beta is a commonly used first-line treatment for RRMS patients, which exerts multiple immunomodulatory functions. It has been reported that IFN-beta treatment reduces Th1 and Th17 cells, the main players initiating and perpetuating the autoimmune attack in MS [61,62,63]. In this study, in vitro human studies with IFN-beta revealed a decrease in the percentage of Th17 cells, although it was not possible to detect changes in the percentage of Th1 lymphocytes after 5 days of co-culture. When VitD3-tolDC were combined with IFN-beta, in vitro results revealed that IFN-beta treatment contributes to the T cell hypo-responsiveness produced by VitD3-tolDC therapy, with the induction of an anti-inflammatory effect caused by a Th17 decrease, as well by an induction of Th2 lymphocytes. 

In the EAE in vivo experiments, although the monotherapy with IFN-beta in mice with clinical signs of EAE was not sufficient to induce a significant reduction of EAE severity, when IFN-beta was combined with antigen-specific VitD3-tolDC-MOG, it was able to potentiate the beneficial effect of these therapeutic cells. Altogether, these results suggest that IFN-beta treatment triggers an anti-inflammatory effect that synergizes together with the tolerogenic functionality of VitD3-tolDC-MOG. As a result, the combined therapy resulted in a powerful clinical effect, significantly reducing EAE disease severity. In fact, in our aggressive EAE model, the efficacy of the combined treatment was so potent that it allowed mice to reach only hind limb paralysis, while mice not receiving treatment (PBS group) or mice treated with IFN-beta or VitD3-tolDC-MOG monotherapies, exhibited different grades of hind limb and forelimb motor alteration.

However, in spite of the presence of in vitro and in vivo evidence demonstrating that VitD3-tolDC are sufficient to decrease T cell proliferation and abrogate EAE disease progression [23,24], the translation of this CTT to the clinical context remains complex due to the chronic inflammatory context of the patients that may affect their functionality [9,18]. In this context and from the abovementioned results, we postulate that the unspecific anti-inflammatory effect of IFN-beta treatment, which has been used for a long time to treat non-aggressive RRMS, could enhance the antigen-specific efficacy of VitD3-tolDC contributing to restore the immune homeostasis.

Our study has focused on the clinical and immunological effect of the combined treatment of VitD3-tolDC with IFN-beta. However, no analysis on the influence of the combined therapy on demyelination was assessed. Future studies should determine if the combination therapy would also induce a decrease in demyelination, what would strongly support the relevance of the combined therapy as a long-term therapeutic strategy for MS patients. In this context, future studies should be addressed to analyze the mechanisms involved in the synergic effect of the combined therapies of VitD3-tolDC plus IFN-beta, as well as with other immunomodulatory or immunosuppressive treatments that have appeared in recent years in the multiple sclerosis arena.

Altogether, our results demonstrate that antigen-specific VitD3-tolDC is an effective therapy able to reduce autoreactive T cell activation. Moreover, the combination of VitD3-tolDC-MOG with IFN-beta treatment resulted in a stronger anti-inflammatory effect and an increased clinical beneficial effect in EAE, suggesting that a combined therapy of antigen-specific cells and immunomodulatory drugs may be a promising new strategy for the treatment of MS patients.

## Figures and Tables

**Table 1:** Percentage of inhibition of allogeneic proliferation in relapsing-remitting multiple sclerosis (RRMS) patients and healthy donors (HD) depending on the treatment.

Condition	RRMS Patients (*n* = 6)		HD (*n* = 6)		*p*-Value
	Mean (%)	SD	Mean (%)	SD	
100%VitD3-tolDC	54.73	9.90	56.14	18.43	0.87
100%VitD3-tolDC + IFN-beta	64.87	8.28	61.14	16.17	0.62
75%VitD3-tolDC: 25%mDC	42.63	23.95	49.29	8.81	0.51
75%VitD3-tolDC: 25%mDC + IFN-beta	57.05	20.09	65.29	12.97	0.39
50%VitD3-tolDC: mDC	21.00	16.00	36.29	10.44	0.14
50%VitD3-tolDC: mDC + IFN-beta	25.00	19.00	45.71	19.54	0.16
25%VitD3-tolDC: 75%mDC	12.00	17.00	19.23	13.99	0.25
25%VitD3-tolDC: 75%mDC + IFN-beta	28.00	6.78	40.86	13.83	0.10

IFN-beta = interferon-beta; Mdc = mature dendritic cells; VitD3-tolDC = vitamin-D3 tolerogenic dendritic cells.

**Table 2:** Percentage of activated CD4+ and CD8+ T cells depending on the therapy.

	Condition	RRMS *n* = 6			HD *n* = 6		*p*-Value
		Mean (%)	SD	*p*-Value	Mean (%)	SD	
CD4 Activated lymphocytes **	mDC	14.03	3.73	-	11.50	8.11	-
	mDC + IFN-beta	12.68	4.92	0.604	9.28	5.95	0.514
	100%VitD3-tolDC	4.95	1.71	**0.003**	5.16	2.60	0.050
	100%VitD3-tolDC + IFN-beta	4.27	1.68	**0.003**	5.25	2.42	0.060
	50% VitD3-tolDC	8.13	3.58	**0.020**	8.01	4.85	0.310
	50% VitD3-tolDC + IFN-beta	10.10	4.77	0.150	7.21	4.63	0.210
CD8 Activated lymphocytes *	mDC	11.33	5.11	-	14.33	8.82	-
	mDC + IFN-beta	10.60	6.07	0.825	13.83	8.37	**0.028**
	100%VitD3-tolDC	3.72	2.12	**0.007**	5.07	4.50	**0.040**
	100%VitD3-tolDC + IFN-beta	3.33	1.56	**0.004**	4.75	2.55	**0.020**
	50% VitD3-tolDC	7.28	1.21	0.087	10.70	7.27	0.450
	50% VitD3-tolDC + IFN-beta	8.70	4.11	0.340	7.62	2.26	0.100

CD4 Activated lymphocytes = CD4^+^HLA-DR^+^ CD38^+^ T lymphocytes; CD8 activated lymphocytes = CD8^+^HLA−DR^+^ CD38^+^ T lymphocytes. HD: healthy donors; IFN-beta: interferon-beta; mDC: mature dendritic cells; RRMS: relapsing-remitting multiple sclerosis; SD: standard deviation; VitD3-tolDC: vitamin D3 tolerogenic dendritic cells. Statistically significant *p* values are marked in bold. * *p* < 0.05, ** *p* < 0.01.

## Supplementary Materials

The following are available online at https://www.mdpi.com/article/10.3390/biomedicines9121758/s1.
