# Supplementary material for: Combined Therapy of Vitamin D3-Tolerogenic Dendritic Cells and Interferon-β in a Preclinical Model of Multiple Sclerosis
Source: Biomedicines. 2021 Nov 24;9(12):1758. doi: 10.3390/biomedicines9121758 (PMC8698295; doi:10.3390/biomedicines9121758)
Supplement: Supplementary file 1 [file biomedicines-09-01758-s001.zip › biomedicines-1411234-supplementary.pdf]

Supplementary Table S1. Clinical and demografic data from multiple sclerosis patients participating in the in vitro study.

ID	MS type	Gender	Age (years)	First symptom of disease (years)	Previous treatment	Whashout period (months)	EDSS	ARR last year
Patient 1	EMRR	W	46	26	IFN-beta<br>NTZ	60	3.0	2
Patient 2	EMRR	W	50	17	IFN-beta	120	2.0	0
Patient 3	EMRR	M	50	22	IFN-beta	72	5.0	0
Patient 4	EMRR	W	26	3	NTZ<br>Fingo	4	3.0	1
Patient 5	EMRR	M	39	2	Naïve	-	1.0	2
Patient 6	EMRR	W	30	0.5	Naïve	-	3.5	1

ARR: Annualized relapse rate; EDSS: Expanded Disability Status Scale; Fingo: Fingolimod; ID: Identification of patients; IFN-beta: interferon-beta; M: male; MS:Multiple Sclerosis; NTZ: natalizumab; W: woman.

Supplementary Table S2. Percentage of lymphocyte subpopulations from Multiple Sclerosis (MS) patients and Healthy Donors (HD) after 5 days co-cultured with mature DC (mDC), 100%VitD3-tolDC or 50%VitD3-tolDC in the presence/absence of IFN-beta

		RRMS		HD	
	Condition	Mean	SD	Mean	SD
CD4 T cells (%)	mDC+IFN-beta	73.90	12.16	63.48	8.57
	100%VitD3-tolDC	72.30	11.71	63.47	9.34
	100%VitD3-tolDC+IFN-beta	72.78	12.83	64.25	9.64
	50% VitD3-tolDC	72.38	12.24	63.57	9.50
	50% VitD3-tolDC+IFN-beta	71.55	11.52	63.92	8.55
CD4 Naïve T cells (%)	mDC	37.95	17.27	55.7	19.11
	mDC+IFN-beta	39.45	14.13	55.85	19.28
	100%VitD3-tolDC	44.38	19.56	62.33	17.68
	100%VitD3-tolDC+IFN-beta	44.85	14.68	59.22	16.58
	50% VitD3-tolDC	40.25	13.28	59.30	17.53
	50% VitD3-tolDC+IFN-beta	43.15	13.59	58.32	16.05
CD4 Central Memory T cells (%)	Mdc	45.40	11.55	31.93	15.58
	mDC+IFN-beta	40.75	7.05	32.33	16.12
	100%VitD3-tolDC	30.88	9.50	25.40	12.04
	100%VitD3-tolDC+IFN-beta	35.40	7.65	27.37	11.56
	50% VitD3-tolDC	39.43	9.18	27.87	13.78
	50% VitD3-tolDC+IFN-beta	37.15	5.67	28.32	12.55
CD4 Effector Memory T cells (%)	mDC	14.65	6.60	10.23	5.49
	mDC+IFN-beta	17.28	9.69	9.95	4.79
	100%VitD3-tolDC	21.33	18.82	10.22	6.42
	100%VitD3-tolDC+IFN-beta	17.28	10.73	11.00	5.88
	50% VitD3-tolDC	18.03	10.14	10.65	5.21
	50% VitD3-tolDC+IFN-beta	17.45	11.46	11.22	5.24
CD4 Effector T cells (%)	mDC	1.97	1.32	2.28	2.83
	mDC+IFN-beta	2.53	1.50	1.82	2.33
	100%VitD3-tolDC	3.47	2.02	2.03	2.16
	100%VitD3-tolDC+IFN-beta	2.47	1.51	1.93	2.52
	50% VitD3-tolDC	2.27	1.33	2.17	2.38
	50% VitD3-tolDC+IFN-beta	2.27	1.54	2.13	2.51
CD4 Activated T cells (%)	mDC	14.03	3.73	11.50	8.11
	mDC+IFN-beta	12.68	4.92	9.28	5.95
	100%VitD3-tolDC	4.95	1.71	5.16	2.60
	100%VitD3-tolDC+IFN-beta	4.27	1.68	5.25	2.42
	50% VitD3-tolDC	8.13	3.58	8.01	4.85
	50% VitD3-tolDC+IFN-beta	10.10	4.77	7.21	4.63
Th1 cells (%)	mDC	41.85	18.21	35.05	9.04
	mDC+IFN-beta	38.25	13.64	31.07	11.30

	100%VitD3-tolDC	36.55	16.08	31.35	5.52
	100%VitD3-tolDC+IFN-beta	32.00	15.77	30.78	10.43
	50% VitD3-tolDC	38.53	10.51	32.55	8.18
	50% VitD3-tolDC+IFN-beta	33.18	13.53	33.38	9.73
Th2 cells (%)	mDC	32.48	4.05	30.93	4.92
	mDC+IFN-beta	36.18	8.49	41.7	10.6
	100%VitD3-tolDC	42.32	4.26	33.50	4.49
	100%VitD3-tolDC+IFN-beta	46.42	5.12	42.43	2.41
	50% VitD3-tolDC	39.66	6.66	39.05	8.66
	50% VitD3-tolDC+IFN-beta	49.30	10.43	43.63	9.63
Th17 cells (%)	mDC	11.40	5.03	14.31	3.04
	mDC+IFN-beta	10.66	5.73	10.51	1.94
	100%VitD3-tolDC	17.92	5.71	18.80	4.82
	100%VitD3-tolDC+IFN-beta	1.51	4.93	15.04	5.65
	50% VitD3-tolDC	13.02	4.72	17.63	3.42
	50% VitD3-tolDC+IFN-beta	11.86	4.72	13.10	2.41
Treg cells (%)	mDC	10.61	7.16	11.02	6.25
	mDC+IFN-beta	10.07	6.65	11.75	7.77
	100%VitD3-tolDC	7.96	5.85	8.54	5.69
	100%VitD3-tolDC+IFN-beta	6.78	5.53	8.69	5.01
	50% VitD3-tolDC	7.86	4.81	10.58	7.05
	50% VitD3-tolDC+IFN-beta	6.85	5.26	9.79	6.70
CD8 T cells (%)	mDC	20.98	8.79	27.82	6.93
	mDC+IFN-beta	21.63	8.68	27.48	7.13
	100%VitD3-tolDC	21.03	8.26	27.03	6.88
	100%VitD3-tolDC+IFN-beta	21.05	7.78	29.72	12.86
	50% VitD3-tolDC	22.75	7.89	27.35	7.27
	50% VitD3-tolDC+IFN-beta	21.90	8.16	27.38	7.16
CD8 Naïve T cells (%)	mDC	43.60	10.45	54.12	8.42
	mDC+IFN-beta	44.80	13.98	55.00	8.12
	100%VitD3-tolDC	52.25	16.05	62.28	7.93
	100%VitD3-tolDC+IFN-beta	53.05	16.08	60.63	6.19
	50% VitD3-tolDC	46.00	16.03	58.53	3.26
	50% VitD3-tolDC+IFN-beta	52.70	15.04	57.60	6.94
CD8 Central Memory T cells (%)	mDC	26.63	10.76	17.08	10.72
	mDC+IFN-beta	22.60	8.15	16.03	9.93
	100%VitD3-tolDC	16.38	8.45	11.13	8.51
	100%VitD3-tolDC+IFN-beta	14.38	9.44	12.07	8.22
	50% VitD3-tolDC	22.98	11.81	13.62	8.59
	50% VitD3-tolDC+IFN-beta	19.13	8.55	13.63	8.21
CD8 Effector Memory T cells (%)	mDC	18.23	7.55	19.50	6.83
	mDC+IFN-beta	20.38	9.57	18.67	5.56
	100%VitD3-tolDC	17.45	8.38	16.88	6.31
	100%VitD3-tolDC+IFN-beta	18.63	8.46	19.02	6.12
	50% VitD3-tolDC	17.68	7.85	17.97	4.04

	50% VitD3-tolDC+IFN-beta	15.80	9.25	19.20	6.12
CD8 Effector T cells (%)	mDC	11.53	10.69	9.78	5.83
	mDC+IFN-beta	12.25	10.03	9.35	5.36
	100%VitD3-tolDC	13.90	12.31	12.23	4.95
	100%VitD3-tolDC+IFN-beta	13.95	12.76	10.50	5.59
	50% VitD3-tolDC	13.35	14.33	10.48	5.59
	50% VitD3-tolDC+IFN-beta	12.38	12.38	10.42	5.29
CD8 Activated T cells (%)	mDC	11.3	5.11	14.33	8.82
	mDC+IFN-beta	10.60	6.07	13.83	8.37
	100%VitD3-tolDC	3.72	2.12	5.07	4.50
	100%VitD3-tolDC+IFN-beta	3.33	1.56	4.75	2.55
	50% VitD3-tolDC	7.28	1.21	10.70	7.27
	50% VitD3-tolDC+IFN-beta	8.70	4.11	7.62	2.26

CD4+ (CD3+CD4+);CD4 Naïve (CD3+CD4+CCR7+CD45RA+);CD4 Central Memory (CD3+CD4+CCR7+CD45RA-); CD4 Effector Memory (CD3+CD4+CCR7-CD45RA+); CD4 Effector (CD3+CD4+CCR7-CD45RA-); CD4 Activated (CD3+ CD4+ HLA-DR+CD38+); Th1 (CD3+ CD4+ CXCR3+ CCR6-); Th2 (CD3+ CD4+CXCR3- CCR6-); Th17 (CD3+ CD4+ CXCR3- CCR6+); CD8+ (CD3+CD8+);CD8 Naïve (CD3+CD8+CCR7+CD45RA+);CD8 Central Memory (CD3+CD8+CCR7+CD45RA-); CD8 Effector Memory (CD3+CD8+CCR7-CD45RA+); CD8 Effector (CD3+CD8+CCR7-CD45RA-); CD8 Activated (CD3+ CD8+ HLA-DR+CD38+); IFN-beta= interferon-beta; mDC= mature dendritic cells; VitD3-tolDC= vitamin-D3 tolerogenic dendritic cells.
